# Use of a scent-detection dog for sea turtle nest monitoring of three sea turtle species in Florida

**DOI:** 10.1371/journal.pone.0290740

**Published:** 2023-09-13

**Authors:** Rebekah J. Lindborg, Pepe Peruyero, Blair E. Witherington

**Affiliations:** 1 Disney’s Animals, Science and Environment, Lake Buena Vista, FL, United States of America; 2 J&K Canine Academy Inc. and Pepedogs™, Alachua, FL, United States of America; 3 Inwater Research Group, Inc., Jensen Beach, FL, United States of America; U.S. Geological Survey, UNITED STATES

## Abstract

Sea turtles are threatened with extinction around the world and rely on sandy beaches for laying their eggs. To protect eggs and locate them for calculation of reproductive success, beach surveyors must find the exact placement of each clutch. Eggs may be buried up to one meter deep under a nest mound several square meters in area. To locate sea turtle eggs, beach surveyors might spend hours searching for these eggs hidden in the sand, especially for difficult-to-locate leatherback (*Dermochelys coriacea)* and green turtle *(Chelonia mydas)* eggs. Scent-detection dogs (*Canis lupus familiaris)* are a novel tool that could provide a means to more accurately identify nests and efficiently locate eggs that need assessment, protection, or relocation. We assessed the effectiveness and feasibility of using a detection dog to locate sea turtle eggs buried in beach sand as compared to the traditional method using human beach surveyors. The detection dog was significantly more accurate in detecting loggerhead sea turtle (*Caretta caretta*) eggs and more efficient (less time spent and fewer holes dug) in assisting with locating the eggs. This case study presents results on the performance of one detection dog only, and additional research is needed with multiple detection dogs and handlers.

## Introduction

Conservation efforts focused on endangered species often require the detection of animals with elusive and cryptic behaviors at different life stages. Population estimates for these species can be uncertain given the difficulty in locating individuals. For sea turtles, the challenge of locating eggs buried in beach sand can result in a high cost from effort and time. Protection of sea turtles in Florida, USA involves locating and assessing the number of nests annually along beaches. These assessments are based on visual cues of sea turtle crawls and nesting sites to determine if a sea turtle nested and where the eggs are located. Locating eggs within a nest allows an accurate reproductive assessment at the conclusion of incubation when visual evidence has been erased by weathering. Unbiased hatchling production estimates should include nests with complete hatching failure, which typically show no external evidence at the end of an incubation period. Determining the precise location of the egg chamber in nests also may be necessary to protect nests from predators [1] or to move nests to more suitable sites [2].

Locating sea turtle eggs is challenging for beach surveyors. Three common sea turtle species found in Florida—the loggerhead (*Carretta caretta*), leatherback (*Dermochelys coriacea*), and green (*Chelonia mydas*) sea turtle–lay their eggs at night. Morning beach surveyors rely on the crawl left behind to determine if the turtle nested or did not nest and where the eggs are located. Non-nesting crawls (often referred to as “false crawls”) are common and occur when a female sea turtle crawls up on the beach to nest but abandons the attempt without laying eggs. Non-nesting crawls may or may not include disturbance of the sand depending on how far in the nesting process the turtle was before abandoning the attempt. Nesting site sizes vary depending on the species and can have eggs that are buried within nest mounds 1 m^2^ for loggerheads, 3 m^2^ for greens, and 9 m^2^ for leatherbacks [3]. Each species of sea turtle also lay their eggs at different depths in the sand, averaging 40 cm for loggerheads, 50 cm for greens, and 60 cm or more for leatherbacks [3]. Clutches sizes for loggerhead sea turtles average 115 eggs, 135 eggs for green sea turtles, and 80 eggs for leatherback sea turtles [3]. During oviposition, sea turtle eggs are coated in cloacal mucus, which may aid in oviposition and possess antimicrobial properties [4, 5].

We assessed the application of domestic dogs (*Canis lupus familiaris*) trained in scent detection, with a trained handler, for assisting in locating buried sea turtle eggs laid by multiple species of turtles along nesting beaches. Detection dogs possess a highly sensitive olfactory system due to their large olfactory epithelium, which contains a greater amount of olfactory sensory neurons as compared to humans [6]. This olfactory system allows dogs to detect odors at a lower threshold and even discriminate among distinct odors while they sniff [6]. Detection dogs have a unique ability to cue in on specific scents that allows the location of objects from great distances, in difficult terrains, and where visual evidence is lacking [6]. In combination with perceptual learning and training, detection dogs can be used to locate specific scents of interest [6]. Researchers have found that detection dogs can locate elusive desert tortoises [7, 8], as well as red-eared slider turtle eggs [9]. Detection dogs commonly outperform human surveyors, including detection of invasive plants [10], bird carcasses [11], and adult desert tortoises [12]. The Padre Island National Seashore sea turtle nesting program has successfully used detection dogs as part of their program to assist in locating Kemp’s Ridley (*Lepidochelys kempii*) nests [13, 14]. Additionally, a pilot study we conducted from 2015–2016 was the first to begin quantifying the effectiveness of a detection dog’s ability to locate sea turtle eggs and the potential of a detection dog as a tool for sea turtle nest monitoring [15]. The current research project builds on this study and assesses the efficacy of using a detection dog for sea turtle nest monitoring, providing a way to locate difficult-to-find sea turtle eggs for more accurate reproductive estimates and increased nest protection. This case study reviews the performance of one detection dog to understand the feasibility of using detection dogs to assist in locating sea turtle eggs.

## Materials and methods

The study protocol was reviewed by, and written approval was received by the Disney’s Animal Care and Welfare Committee (IR1808). The field research was approved and permitted by the Florida Fish and Wildlife Conservation Commission (Marine Turtle Permit #071 & 260). The study site was a 7 km stretch of beach in Indian River County, Florida, USA near Disney’s Vero Beach Resort. There, we surveyed nests from loggerhead, green, and leatherback sea turtles using either the detection dog/handler team or human surveyors alone. The surveys spanned peak nesting, which for loggerheads in this region occurs in June and July, and for green turtles occurs in July and August [16]. Mean annual nesting density for the study site was 1280 nests, with a mean of 945 loggerhead nests, 321 green nests, and 9 leatherback nests laid annually (Lindborg, *personal observation*). We aimed to have a minimum sample size of 250 nests sampled by the detection dog team and 250 nests sampled by the human surveyors (power analysis for two-group independent sample t-test, alpha = 0.05, beta = 0.20, effect = 0.10). A subset of nests laid along the study area were sampled following the Florida Fish and Wildlife Conservation Commission (FWC) Marine Turtle Conservation Handbook guidelines [17].

### Training the detection dog

The detection dog, a female, terrier mix named Dory, was hand-selected and trained by Pepe Peruyero (hereafter referred to as “lead trainer”) of Pepedogs^™^, located in Gainesville, Florida, USA. At the time of selection, Dory was a 2–3 year old, 18-kg, dog that displayed key dispositional characteristics qualifying her for scent-detection work, including high focus and high drive [6, 18, 19]. Dory demonstrated desirable qualities during the selection process and had certain physical characteristics necessary for the type of fieldwork she would be performing, including short fur and a medium body size for increased endurance in hot field conditions [6].

The lead author was selected as the handler for the detection dog throughout the study period (hereafter referred to as “dog handler”). It is important to note that this study focused on the training and use of one detection dog and handler (hereafter referred to as the “dog/handler team”), and the results only draw conclusions based on the performance of this one dog only and not on detection dogs in general. Initial training of the detection dog occurred with the lead trainer in Gainesville, Florida, USA. Subsequent maintenance training continued in Orlando, Florida and Wabasso Beach, Florida where we used the detection dog for field-based conservation work in 2017 and 2018 (two sea turtle field seasons). Detailed training methods, including imprinting, simulation, controlled and blind hides, and deployment can be found in the supporting information S1 Appendix. The target odor we chose for egg detection was sea turtle cloacal mucus, which is the fluid female turtles produce that surrounds the eggs following oviposition. We selected cloacal mucus because it provided a means of easily gathering the scent of a sea turtle nest without collecting or sacrificing eggs. We also hypothesized that scent from cloacal mucus would fade as the mucus dried, making the scent strongest at fresh nests. During a pilot study conducted in 2015 and 2016, the detection dog did not present the final response on week-old nests [15]. We collected cloacal mucus from nesting loggerhead and leatherback sea turtles using sterile swabs, which were then frozen to preserve the scent until they could be used for training purposes.

### Data collection methods

#### Detection dog accuracy

**Fig 1:**
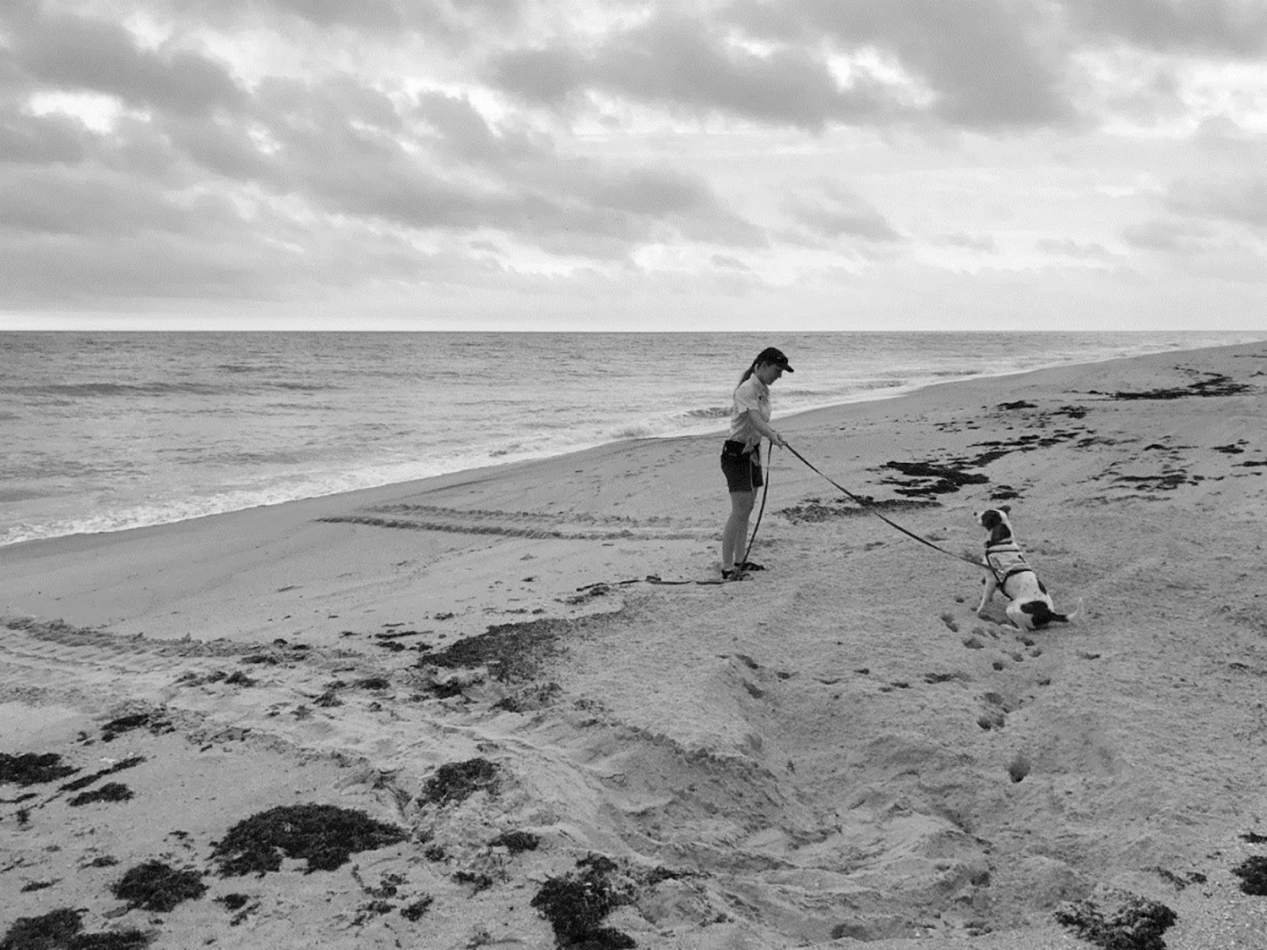
Detection dog final response behavior at green turtle nesting site. The detection dog presenting a final response (sit) at a green turtle nesting site. The nesting site encompassed the disturbed area, with clear signs of sand being thrown into a mound between the up-crawl and the down-crawl. The eggs were located approximately 55 cm under sand where the dog’s nose is pictured. Printed under a CC BY license, with permission from Disney Worldwide Services, Inc., original copyright 2017.

We used the dog/handler team to assist in locating eggs within a subset of sea turtle nests from May-August in 2017 and 2018. The study used the Clutchkeeper iPad application [20] to systematically select every *n*th nest (e.g., every 8th loggerhead nest, every 2nd green nest) for sampling following FWC Marine Turtle Conservation Handbook guidelines [17]. In 2018, we used the detection dog team on a greater number of nests outside of the sampling scheme for extra training. Use of the detection dog included the dog/handler team where the dog handler always maintained the detection dog on a leash and harness and traversed the beach on an all-terrain vehicle (ATV) between each crawl. The dog handler’s role included transporting the dog to each presumptive nest location (identified by tracks and sand disturbance) and interpreting the detection dog’s behavior to determine the clutch location represented by the final response. Detailed training methods of the dog handler can be found in the supporting information S1 Appendix. We used wind direction to dictate where to begin the detection dog’s search, so the dog was always started downwind if there was enough discernable wind to determine this direction. We determined the detection dog was on odor (detecting scent) when her nose was near the ground/sand surface and moving back and forth rapidly. During this time, the dog handler paid close attention to the detection dog’s nose position to determine the precise location of the clutch following the final response (sit) behavior (Fig 1). After the detection dog’s final response, a survey flag was placed in the sand to mark the location. The dog handler would then dig at the final response location to locate the eggs buried in the sand.

To assess the effectiveness of a detection dog for assisting with locating sea turtle eggs, we collected data on specific measures that typically define the success of scent-detection training, including measures of success and effort. Measures of success included accuracy, specificity, and sensitivity. Accuracy was measured as the horizontal distance from the detection dog’s initial final response location to the confirmed center of the clutch. Other measures of success were collected by recording the number of true positives (presented final response at target stimulus), false positives (presented final response at non-target stimulus), true negatives (did not present final response at non-target stimulus), and false negatives (did not present final response at target stimulus) [6] (Table 1).

**Table 1:** Possible final responses that can be expected from the detection dog and whether or not the target stimulus is present at response location.

	Detection Dog’s Response	
What is Physically Present at Response Location	Target Stimulus Present	Target Stimulus Not Present
**Target Stimulus Present **	True Positive	False Negative
**Target Stimulus Not Present **	False Positive	True Negative

These parameters provided information on the detection dog’s sensitivity, the proportion of correct detections calculated using the formula “true positive / (true positive + false negative)” and specificity, the proportion of false positives calculated using the formula “true negative/(false positive + true negative)” [6]. Sea turtle crawls included both nests and non-nesting crawls (hereafter referred to as “false crawls”). False crawls are tracks, often with sand disturbed, but do not include eggs. We recorded any false positives (the detection dog presenting a final response at a false crawl) and false negatives (the detection dog not presenting a final response at a nest) if they occurred. Measures of effort included the search time spent locating the clutch with the dog/handler team, and the number of holes dug to locate the clutch with the dog/handler team. Search time included when the dog handler first gave the command to the detection dog to search the nesting site, the detection dog searching the nesting site, the final response behavior and reward/reinforcement from the handler, the handler placing the detection dog back in her crate, and the dog handler digging to search for the eggs. The search time ended when the handler located the top egg in the clutch. We recorded potential predictors of the detection dog’s accuracy, including clutch depth (m), sea turtle species, wind speed (m/s) (using the Weatherhawk Skymate thermos-wind meter model SM-18) and wind direction, sand surface temperature (°C) (using a Pasco wireless temperature sensor probe model PS-3201), ambient temperature (°C), humidity, rainfall, and the number of hours the dog had worked. We set up a Hobo weather station near the study location to collect data on relative humidity, ambient temperature, and precipitation, which recorded these weather data every 30 minutes. To test the predictors that might affect the detection dog’s accuracy, a multiple regression was performed to create a model selection of these factors.

#### Human accuracy

Human surveyors collected data on a subset of loggerhead, green turtle, and leatherback nests along the same 7-km study beach surveyed with the detection dog team, but on different days, and also used an ATV as a mode of transportation between crawls. We collected data on a total of 14 different human surveyors throughout the course of this study whose level of expertise varied from one year of sea turtle nest monitoring to over 30 years and all had been trained and permitted through FWC. To facilitate data collection, we used the Clutchkeeper iPad app [20] for all nests assessed by human surveyors during April-September in 2017 and 2018. The iPad application selected nests following every *n*th nest marking scheme. Human surveyors collected data regarding accuracy, efficiency, and effort.

Data collected included sea turtle species, search time (in seconds) spent locating the eggs (time included from the start of searching until the top egg was located), the number of holes dug to locate the eggs, clutch depth (m), and the horizontal distance between the first hole dug (first guess) to the center of the clutch location (m). For human surveyors, the search time included when they started assessing the nesting site (looking for signs to determine egg location visually), the surveyors digging to find the eggs, and search time ended when they located the top egg.

### Practical measure of efficiency

In addition to accuracy and efficiency comparisons of human surveyors and the detection dog team, we tested a method hypothesized to maximize efficiency in clutch location for nest marking. This method involved marking the nesting site (cordoning it off during incubation) at the site of the detection dog’s final response without digging to confirm the clutch location. Instead, the dog handler marked the nest at the location of the detection dog’s final response and inventoried the nest three days after signs of hatchling emergence, or after 70 days, to verify clutch location and measure hatching success. We centered the stakes around the final response location to reduce the likelihood that the stakes would be accidentally placed through the clutch. The same data as described above was collected by human surveyors after the nest emerged or at day 70 if no emergence was seen.

### Analyses

We ran all analyses using IBM SPSS statistical software version 21.0. To compare the detection dog’s accuracy and efficiency for locating sea turtle nests with that of the human control, we performed an independent samples t-test statistical analysis. Data from nests of all species were lumped and also analyzed individually to determine overall accuracy and effects from species. When parametric tests were not appropriate, we used a Mann-Whitney U non-parametric alternative.

We used multiple regression to test the predictors that might affect the detection dog’s accuracy, including clutch depth (m), sea turtle species, wind speed (m/s) and wind direction, sand surface temperature (°C), ambient temperature (°C), humidity, the number of hours the detection dog worked per day, and rainfall on the detection dog’s accuracy.

We checked the assumptions of the multiple regression and multicollinearity was not violated. Additionally, we removed three extreme outliers out of 521 data points following procedures outlined using Mahalanobis Distance values [21]. While inspecting the Normal Probability Plot (P-P) of the Regression Standardized Residual, we found that the residuals appeared normally distributed and given the large sample size we used regression analysis [22, 23].

### Detection dog welfare

The health and welfare of the detection dog were critical to the success of this study. The detection dog was under the care of the Disney’s Animals, Science and Environment veterinarian team. We took into consideration the physical welfare of the detection dog at all times, ensuring the dog was always properly hydrated and had access to shade and a cooling fan. Signals regularly monitored for heat exhaustion included gum discoloration, excessive panting, and lethargy. We outfitted the ATV used to transport the detection dog with a soft crate to allow air circulation, a cooling mat inside the crate, a fan to increase airflow, and an adjustable umbrella to keep the detection dog in the shade during transportation. A Hobo data logger on the collar of the detection dog monitored the temperature every 30 minutes during the time the detection dog was working, and sand surface temperature was recorded at each sea turtle crawl location. Our protocol was to remove the detection dog from the beach immediately and take her to an air-conditioned building should there be any sign of critical heat stress, and to leave the beach if she no longer showed interest in working.

## Results

Annual average nest counts within the 7-km beach during the study period (2017–2018) revealed an active beach with 957 loggerhead nests, 374 green turtle nests, and 7 leatherback nests (R. Lindborg, *personal observation*). We sampled 560 nests using the detection dog (262 nests in 2017 and 298 nests in 2018; Table 2). We also sampled 113 false crawls using the detection dog (50 in 2017 and 63 in 2018). Of the 560 nests sampled using the detection dog, the clutch could not be located in 5.7% of the sampled nests.

**Table 2:** Total number of sampled nests per sampling year and per species for the detection dog and human surveyors, including nests where the clutch was not located.

Species	Sampling year	Dog *n*	Human *n*
Loggerhead	2017	158	41
	2018	285	153
	2017–2018	443	194
Green turtle	2017	102	58
	2018	9	4
	2017–2018	111	62
Leatherback	2017	2	-
	2018	4	-
	2017–2018	6	-
TOTAL	2017	262	99
	2018	298	157
	2017–2018	560	256

Concurrent with nests sampled using the detection dog team, human surveyors sampled a total of 256 nests (99 in 2017 and 157 in 2018; Table 2). Of the 256 nests sampled using human surveyors, the clutch was not successfully located in 14.8% of the sampled nests. Because the data were positively skewed (right skewed) with zero values, we transformed the data using a Log10 + 1 transformation so the data would fit the normality assumptions needed for a parametric test [21]. We used two tests of normality to check the results of the data transformation, the Kolmogorov-Smirnov and the Shapiro-Wilks tests.

### Measures of success: accuracy, specificity, and sensitivity

The mean accuracy of the detection dog, as measured by horizontal distance from the dog’s final response location to the center of the clutch, across both years and total nests sampled was 0.17 ± 0.21 m (n = 521; Table 3). For loggerhead nests, the detection dog had a mean accuracy of 0.12 ± 0.14 m (n = 431) and a mean accuracy of 0.39 ± 0.35 m (n = 86) for green turtles (Table 3). For leatherback sea turtle nests, the mean accuracy of the detection dog was 0.42 ± 0.11 m (n = 4; Table 3). The detection dog correctly identified sea turtle nests (correct true positive final response, no false negative final response) 100% of the time (n = 560). Additionally, the detection dog correctly identified false crawls (correct true negative final response, no false positive final response) 100% of the time (n = 113).

**Table 3:** Accuracy of the detection dog as compared to human surveyors.

		Detection dog accuracy				Human surveyor accuracy			
Species	Sampling year	*n*	$\bar{x}\pm SD\;(m)$	$\tilde{x}$	Range	*n*	$\bar{x}\pm SD\;(m)$	$\tilde{x}$	Range
All Nests Sampled	2017	229	0.25 ± 0.27	0.19	1.70	81	0.28 ± 0.22	0.30	0.96
	2018	292	0.10 ± 0.11	0.06	0.80	137	0.23 ± 0.18	0.20	0.88
	2017&2018	521	0.17 ± 0.21	0.09	1.70	218	0.25 ± 0.20	0.22	0.96
Loggerhead	2017	150	0.18 ± 0.18	0.15	0.85	40	0.21 ± 0.18	0.20	0.67
	2018	281	0.09 ± 0.10	0.06	0.79	136	0.23 ± 0.18	0.20	0.87
	2017&2018	431	0.12 ± 0.14	0.07	0.85	176	0.22 ± 0.18	0.20	0.88
Green turtle	2017	77	0.39 ± 0.29	0.19	1.70	41	0.35 ± 0.23	0.35	0.96
	2018	9*	0.34 ± 0.10	0.34	0.69	1*	-	-	-
	2017&2018	86	0.39 ± 0.35	0.24	1.70	42	0.35 ± 0.23	0.35	0.96
Leatherback	2017	2*	0.50 ± 0.23	0.50	0.20	-	-	-	-
	2018	2*	0.34 ± 0.17	0.34	0.05	-	-	-	-
	2017&2018	4*	0.42 ± 0.11	0.38	0.29	-	-	-	-

Mean accuracy (horizontal distance in meters between initial estimate and center of true clutch position, $\bar{x}$ ± SD), median ($\tilde{x}$), and range for the detection dog team and human surveyors represented by individual sampling years. Mean accuracy, median, and range are also represented by species, including loggerhead, green turtle, and leatherback nests. The sample size (*n*) is indicated by each sampling year with specific sampling years (*) having too small of sample size to draw any significant conclusions. These data represent information collected only from nests where the clutch was located.

#### Detection dog and human accuracy

**Fig 2:**
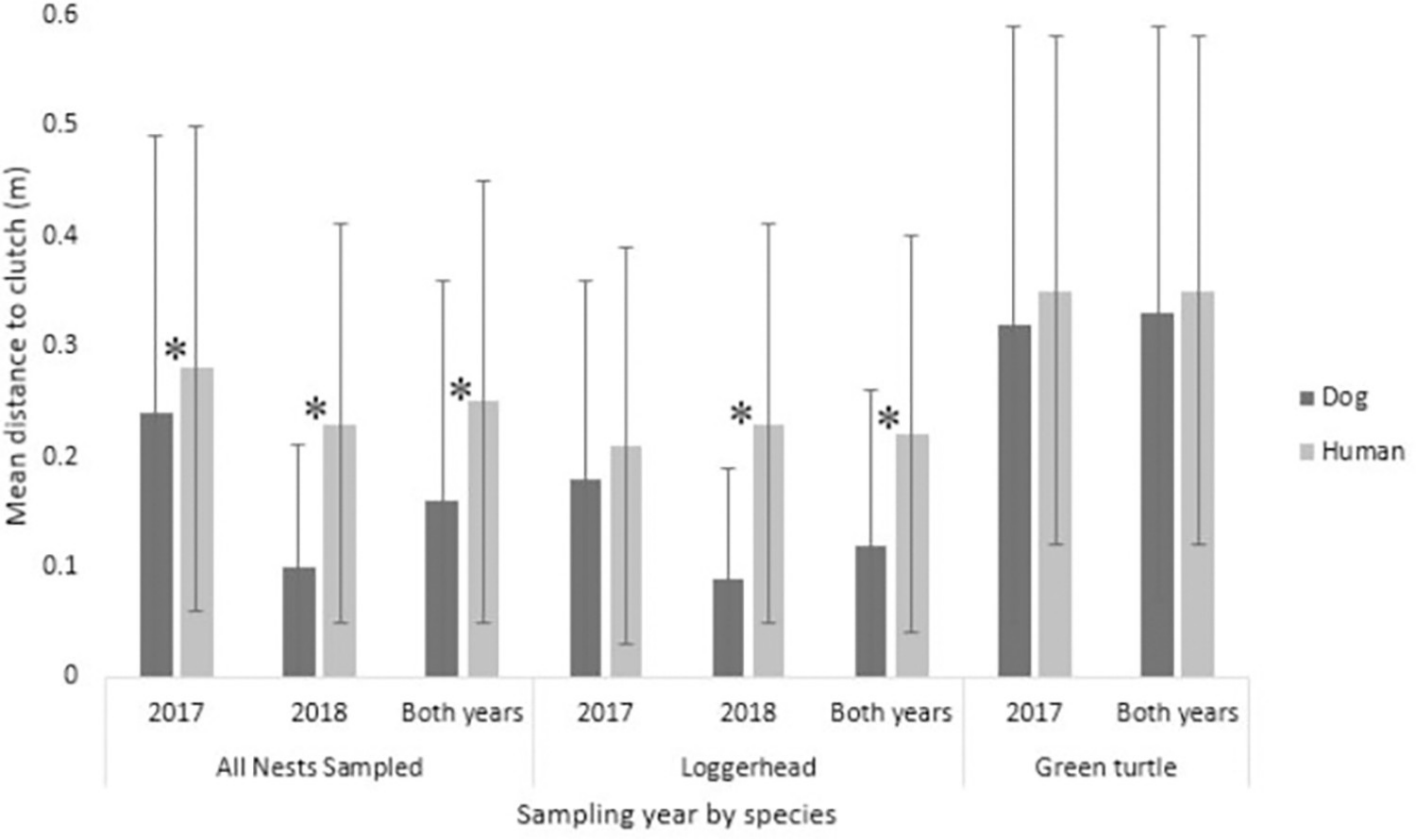
Accuracy of the detection dog as compared to human surveyors. Mean accuracy (distance to clutch) by sampling years and species (loggerhead and green turtle) comparing the detection dog with the human surveyors. Statistically significant differences (α = 0.05) between the detection dog’s accuracy and human surveyors’ accuracy are represented by the star symbol (*). All nests combined 2017 (Z_309_ = -2.070, *p* = 0.038), 2018 (Z_428_ = -8.218, *p*<0.001), both years (Z_738_ = -6.450, *p*<0.001). Loggerhead 2017 (Z_189_ = -1.105, *p* = 0.269), 2018 (Z_416_ = -8.672, *p*<0.001), both years (Z_606_ = -7.266, *p*<0.001). Green turtle 2017 (Z_111_ = -1.130, *p =* 0.258), both years (Z_121_ = -1.038, *p* = 0.299).

The mean accuracy for the human surveyors (distance from the first hole dug to clutch center) for both years combined and total nests sampled was 0.25 ± 0.20 m (n = 218; Table 2). For loggerhead nests, the human surveyors had a mean accuracy of 0.22 ± 0.18 m (n = 176) and a mean accuracy of 0.35 ± 0.23 m (n = 42) for green turtles (Table 3). Human surveyors did not sample any leatherback nests during the study period because of the low nesting density at the study site. The Log10 + 1 transformation of the data did not improve the normality of the accuracy, search time, or number of holes dug data; therefore, we used a Mann-Whitney U non-parametric alternative statistical analysis to compare these accuracy and effort data between the detection dog and human surveyors. The detection dog showed better accuracy than human surveyors for total nests sampled in both years (Z738 = -6.845, p<0.001; Table 3, Fig 2). The accuracy was also better for the detection dog than the human surveyors both years when comparing only loggerhead nests (Z606 = -7.66, p<0.001) but was not significantly different when comparing only green turtle nests (Z121 = -1.038, p = 0.299; Table 3, Fig 2).

When comparing the final response location of the detection dog (n = 521) to the initial guess made (first hole dug) by a human surveyor (n = 218) with total nests sampled, the detection dog correctly presented a final response within 30 cm of the clutch location at a rate of 84.1% while the human surveyors were within 30 cm of the clutch location 69.1% of occurrences. This difference in accuracy between the detection dog and the human surveyors was significant (binominal two population test, Z742 = 4.628, p<0.001). Taking into consideration median search area (median accuracy) with total nests sampled (Table 3), the detection dog’s final response was median 0.09 m from the center of the clutch and the human surveyors’ first guess was median 0.22 m from the center of the clutch, which means the dog was 83% more accurate in area than the human surveyors.

### Measures of effort: Search time and holes dug

**Fig 3:**
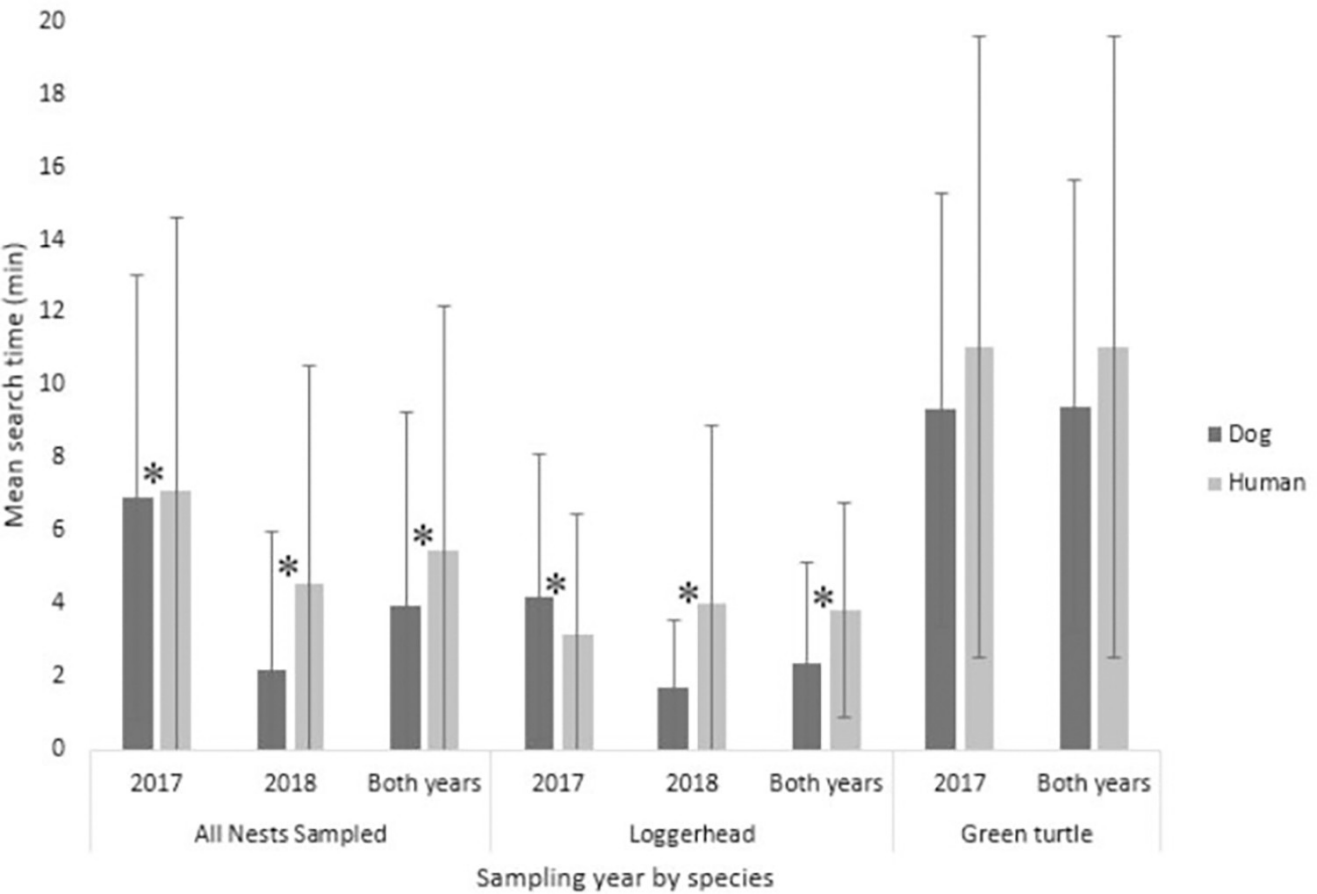
Search time of the detection dog as compared to human surveyors. Mean search times by sampling years and species (loggerhead and green turtle) comparing the detection dog with the human surveyors. Statistically significant differences (α = 0.05) between the detection dog’s search times and human surveyors’ search times are represented by the star symbol (*). All nests sampled 2017 (Z_255_ = -2.031, *p* = 0.042), 2018 (Z_428_ = -6.973, *p*<0.001), both years (Z_684_ = -3.783, *p<*0.001). Loggerhead 2017 (Z_135_ = -3.869, *p<*0.001), 2018 (Z_416_ = -7.324, *p<*0.001), both years (Z_552_ = -4.662, *p*<0.001). Green turtle 2017 (Z_113_ = -0.023, *p* = 0.982), both years (Z_122_ = -0.021, *p* = 0.983).

The mean search time for the detection dog team with all sampled nests was 3.69 ± 4.64 min (n = 463; Table 4). For loggerheads, the detection dog team had a mean search time of 2.35 ± 2.77 min (n = 377), and a mean search time of 9.18 ± 5.76 min for green turtles (n = 82; Table 4). For leatherback sea turtle nests, the mean search time for the detection dog team was 16.00 ± 14.02 min (n = 4; Table 4). The mean search time for human surveyors with all sampled nests was 5.06 ± 6.05 min (n = 216; Table 4). For loggerheads, the human surveyors’ mean search time was 3.76 ± 4.56 min (n = 174), and for green turtles the mean search time was 10.45 ± 8.31 min (n = 41; Table 4). Using a Mann-Whitney U statistical analysis, the detection dog team showed shorter search times than human surveyors for all sampled nests combined in both years (Z684 = -3.783, p<0.001; Table 4, Fig 3). The search times were also shorter for the detection dog team than the human surveyors when comparing loggerhead nests (Z552 = - 4.662, p<0.001) but was not significantly different when comparing green turtle nests (Z122 = - 0.021, p = 0.983; Table 4, Fig 3).

**Table 4:** Search time of the detection dog as compared to human surveyors.

		Detection dog search time (min)				Human surveyor search time (min)			
Species	Sampling year	*n*	$\bar{x}\pm SD$	$\tilde{x}$	Range	*n*	$\bar{x}\pm SD$	$\tilde{x}$	Range
All Nests Sampled	2017	171	6.42 ± 5.47	4.95	36.14	81	7.10 ± 7.53	3.52	27.38
	2018	292	2.08 ± 3.11	1.17	34.76	135	4.55 ± 6.00	1.98	35.05
	2017&2018	463	3.69 ± 4.64	1.98	36.77	216	5.06 ± 6.05	2.79	35.05
Loggerhead	2017	96	4.23 ± 3.90	3.52	35.14	40	3.17 ± 3.32	1.75	12.72
	2018	281	1.72 ± 1.90	1.12	18.53	134	3.94 ± 4.87	2.38	35.25
	2017&2018	377	2.35 ± 2.77	1.39	35.77	174	3.76 ± 4.56	2.30	35.25
Green turtle	2017	73	9.33 ± 5.95	8.23	34.25	41	10.50 ± 8.31	8.00	27.00
	2018	9*	7.90 ± 3.77	8.00	10.85	-	-	-	-
	2017–2018	82	9.18 ± 5.76	8.11	34.25	41	10.45 ± 8.31	8.00	27.00
Leatherback	2017	2*	5.42 ± 0.24	5.42	0.17	-	-	-	-
	2018	2*	21.60 ± 4.82	21.60	16.82	-	-	-	-
	2017&2018	4*	16.00 ± 14.02	11.88	29.75	-	-	-	-

Mean search times ($\bar{x}$ ± SD), median ($\tilde{x}$), and range in decimal minutes for the detection dog team and human surveyors represented by individual sampling years. Mean search time, median, and range are also represented by species, including loggerhead, green turtle, and leatherback nests. The sample size (*n*) is indicated by each sampling year with specific sampling years (*) having too small of sample size to draw any significant conclusions. These data represent information collected only from nests where the clutch was located.

The second measure of effort recorded was the number of holes dug to locate the eggs. With all species combined, the percentage of nests with one hole dug using the detection dog team was 61.6% as compared to 42.9% with human surveyors (Table 5). For loggerhead nests, the percentage of nests with one hole dug was 69.3% using the detection dog team and 49.5% with human surveyors (Table 5). For green turtle nests, the dog/handler team dug one hole to locate the eggs 25.9% of the time while human surveyors dug one hole to locate the eggs 19.1% of the time (Table 5). Using a Mann-Whitney U statistical analysis, the detection dog nests had fewer holes dug than the human surveyors for all three species combined in both years (Z732 = -4.875, p<0.001; Table 5). The number of holes dug was also fewer for the detection dog nests than the human surveyor nests when comparing loggerhead nests (Z601 = -4.754, p<0.001) but was not significantly different when comparing green turtle nests (Z125 = -1.263, p = 0.206; Table 5).

**Table 5:** Proportion of holes dug comparing the detection dog team and the human surveyors by individual sampling year.

		Detection dog holes dug						Human surveyor holes dug					
Species	Year	*n*	1	2	3	4	≥5	*n*	1	2	3	4	≥5
All Nests Sampled	2017*	222	41.1%	23.7%	10.3%	7.59%	17.4%	81	35.2%	12.5%	13.6%	9.09%	29.5%
	2018*	290	77.4%	12.0%	3.77%	2.40%	4.45%	137	48.9%	25.2%	7.91%	7.19%	10.8%
	2017&2018*	512	61.6%	17.1%	6.59%	4.65%	10.1%	218	42.9%	19.9%	9.96%	7.79%	17.5%
Loggerhead	2017	146	48.6%	24.7%	10.3%	5.4%	11.0%	40	55.0%	7.50%	12.5%	15.0%	10.0%
	2018*	281	80.1%	11.7%	3.91%	2.14%	2.14%	137	48.9%	25.2%	7.91%	7.19%	10.8%
	2017&2018*	427	69.3%	16.2%	6.09%	3.28%	5.15%	177	49.5%	20.9%	8.79%	8.79%	12.1%
Green turtle	2017	76	27.6%	22.4%	7.89%	11.8%	30.3%	41	19.1%	17.0%	14.9%	4.26%	44.7%
	2018	9	11.0%	22.0%	0.0%	11.0%	55.5%	0	-	-	-	-	-
	2017&2018	85	25.9%	22.4%	7.06%	11.8%	32.9%	41	19.1%	17.0%	14.9%	4.26%	44.7%

The proportion of holes dug are also represented by species, including loggerhead and green turtle. The sample size (*n*) is indicated by each sampling year. Years, where a statistically significant difference (α = 0.05) was found using a Mann-Whitney U between the number of holes dug using the detection dog as compared to the human surveyors, are represented with an asterisk (*). All nests sampled 2017 (Z_304_ = -2.23, *p* = 0.025), 2018 (Z_428_ = -5.903. *p*<0.001), both years (Z_732_ = -4.870, *p*<0.001). Loggerhead 2017 (Z_185_ = -0.030, *p =* 0.976), 2018 (Z_415_ = -6.710, *p*<0.001), both years (Z_601_ = -4.754, *p*<0.001). Green turtle 2017 (Z_116_ = -1.519, *p* = .129), both years (Z_125_ = -1.263, *p* = 0.206).

### Predictors of accuracy

We used a multiple regression to predict the effects of clutch depth (m), sea turtle species, wind speed (m/s) and wind direction, sand surface temperature (°C), ambient temperature (°C), humidity, and the number of hours the detection dog worked in a day. Throughout the study period, no rainfall occurred the night before or while the detection dog was working so we removed rainfall as a predictor from the analysis. The Pearson correlation table presented with the analysis showed significant, positive correlations between accuracy and sea turtle species (r147 = 0.465, p<0.001), clutch depth (r147 = 0.350, p<0.001), and ambient temperature (r147 = 0.159, p<0.001), and a significant, negative correlation between accuracy and wind direction (r147 = -0.445, p<0.001; Table 6). We found a significant regression equation (F8,139 = 10.128, p<0.001), with an R2 of 0.370. The results showed that sea turtle species (β = 0.325, p = 0.001) and wind direction (β = -0.396, p<0.001) significantly predicted the detection dog’s accuracy (Table 7).

**Table 6:** Results of the correlation analysis presented with the multiple regression showing the correlation between accuracy (response variable) and the predictor variables shown below.

Variables	r-value	*p-*value
Sea turtle species*	0.465	0.001
Clutch depth*	0.350	0.001
Sand temperature	0.041	0.181
Wind speed	0.038	0.201
Wind direction*	-0.445	0.001
Ambient temperature*	0.159	0.001
Humidity	-0.060	0.100
Number of hours worked	0.064	0.073

The r-values show the strength and direction of the correlation while the p-values show the significance of the correlation. Statistically significant correlations (α = 0.05) are denoted by an asterisk (*).

**Table 7:** Results of the standard multiple regression assessing the effects of the below predictors (variables) on the detection dog’s accuracy.

Variable	Coefficient (β)	t-value	*p*-value
Sea turtle species*	0.325	3.299	0.001
Clutch depth	0.111	1.178	0.241
Sand temperature	-0.051	-0.526	0.599
Wind speed	0.046	0.614	0.540
Wind direction*	-0.396	-5.675	0.001
Ambient temperature	0.043	0.397	0.692
Humidity	0.045	0.473	0.637
Number of hours worked	0.001	0.009	0.993

The largest β values below indicate which variable provides the strongest contribution to explaining the effect on accuracy. Both species and wind direction (denoted by an *) have the largest β values and the only statistically significant *p*-values less than 0.05.

### Practical measure of efficiency

For the nests where we marked the final response location of the detection dog without digging to confirm the clutch location, we sampled a total of 37 nests. Of those 37 nests sampled, human surveyors inventoried 28 nests two months following oviposition after they observed signs of hatchling emergence. The human surveyors inventoried the remaining 9 nests in the sample 70 days after oviposition following FWC Marine Turtle Conservation Handbook guidelines because we observed no signs of hatchling emergence [17]. We located a total of 36 of the 37 nests with a mean of 1 hole dug to locate the eggs and a mean of 1.25 ± 3.23 min spent locating the eggs. The detection dog’s mean accuracy for these sampled nests was 0.11 ± 0.12 m from her final response location to the center of the clutch location. The detection dog’s mean search time (the time the dog spent searching for the nest before presenting final response) was 29.5 ± 9.9 seconds. The mean effort expended for these sampled nests, including the time spent using the detection dog to search for the eggs and the time spent searching for the eggs during inventory was 1.71 ± 3.18 min.

### Physical welfare measures

The mean ambient temperature the detection dog experienced (from the data collected on the Hobo data logger) was 28.8 ± 4.2°C and the mean sand temperature was 26.9 ± 5.2°C. In 2017, the mean ambient temperature was 28.8 ± 4.7°C and mean sand temperature was 27.0 ± 5.2°C. In 2018, the ambient mean temperature was 28.8 ± 3.4°C and the mean sand temperature was 26.8 ± 5.3°C. The maximum ambient temperature the detection dog experienced for a brief period of time (<30 min) in 2017 was 40.1°C, which occurred at the end of June, while the maximum ambient temperature the detection dog experienced in 2018 was 35.2°C. Throughout the entire study period from 2017 and 2018, the detection dog did not experience any symptoms of heat exhaustion and did not have to be removed from the beach prior to the end of the survey because of heat exhaustion. In 2017, there was one instance where the detection dog did end the survey early because she was showing signs of fatigue from working but no signs of heat exhaustion. In this instance, she laid down after presenting the final response at a nest and voluntarily chose to end the session. During this instance, the detection dog had been working for just over six hours, although the mean time spent working daily was 2.25 ± 0.98 hours. This instance was the only time the detection dog left the beach before completing the survey during the entire study period, and this instance only resulted in two nests not being sampled using the detection dog.

## Discussion

The detection dog outperformed human surveyors in measures of success and effort, demonstrating the potential of using detection dogs as a tool for assisting in locating sea turtle eggs for conservation. By reducing the number of holes dug and the search time involved locating the eggs, the dog/handler team significantly reduced the amount of effort spent searching for sea turtle eggs. The detection dog was significantly more accurate in pinpointing the location of the eggs, which reduced the effort of digging to locate the eggs. This reduction in effort also reduced the overall time spent on the beach by surveyors, limiting the time spent out during the heat of the day, especially in the busy months of June and July (months with the most sea turtle nests) when temperatures were highest.

### Species effect

We found evidence of a sea turtle species’ effect on the detection dog’s accuracy. Accuracy was lowest for green turtle nests and was not significantly different from the accuracy of our human surveyors. Green turtle nesting sites are larger than loggerhead nests, and the eggs are buried deeper, on average, making the eggs more challenging to locate even for human surveyors [3]. However, it is likely that the dog’s accuracy at green turtle nests was under-represented during the season when the detection dog was most experienced. The detection dog sampled 102 green turtle nests in 2017 but only 9 green turtle nests in 2018 because of the biennial cycle that green turtles exhibit during nesting seasons [3]. The detection dog’s accuracy improved with loggerhead nests from a mean of 0.18 m in 2017 (n = 150) to a mean of 0.09 m in 2018 (n = 281), which was likely a result of the detection dog being able to continue to sample a large number of loggerhead nests. The low green turtle nesting in 2018 might have had two effects: a reduction of statistical power in the comparison to human surveyors, and fewer opportunities for the dog to improve her accuracy with experience at green turtle nests. Thus, the species effect we observed may have been a training effect.

Additionally, it is unclear if there was any effect regarding the cloacal mucus odor the detection dog was trained to detect. Cloacal mucus was collected from leatherback and loggerhead sea turtles for training purposes. The detection dog was able to detect green turtle cloacal mucus odor after training only with mucus scent from other species, but it is unclear if variations in the odor between species influenced the accuracy and efficiency of the detection dog for detecting green sea turtle eggs. More research would need to be conducted to understand if there is a discernable difference in the cloacal mucus odor produced by different sea turtle species.

Given the progression of accuracy seen with loggerhead nests, we expect that the detection dog would have shown similar progress with green turtle nests as her experience with these nests continued in subsequent years. Although the sample size of leatherback nests was too small to allow conclusions for this species, we hypothesize that the detection dog would also exhibit greater accuracy with leatherback nests provided that more nests were sampled in subsequent years. Other studies have also noted the importance of maintenance training for detection dogs by providing frequent opportunities for the detection dog to practice on target odors [24, 25], and that detection dogs tend to increase in accuracy over time as they learn to better discriminate the target odor [7, 26–28]. Although the low nesting density did not allow for human comparisons with leatherback nests, leatherback eggs are notoriously difficult to locate and human surveyors can spend multiple hours searching for the eggs and still not find them (Lindborg, *personal observation*). When leatherback eggs are not located, the entire nesting site must be marked off and monitored for the duration of the incubation period for the possibility of seeing the hatching emergence [17], increasing both the effort expended throughout incubation and the potential sampling bias from missed emergence evidence. Because the detection dog displayed the potential for reducing the time and effort spent locating leatherback eggs, with more experience (a larger sampling of leatherback and green turtle nests) and development of her skills, we predict that her accuracy with these species would progress to a level similar to loggerhead nests.

### Covariates

There was a wide range of environmental conditions the detection dog experienced on the beach, but few of the tested factors were found to affect the detection dog’s accuracy. Some covariates tested were exclusive to sea turtle monitoring, specifically, sea turtle species effects and clutch depth as factors hypothesized to affect the detection dog’s accuracy. We chose these factors because each sea turtle species has large differences in nest site preparation, including the amount of sand thrown and the size of the disturbed area. Similarly, we chose to measure clutch depth because each species tends to bury their eggs at different depths in the sand [29], potentially making it more difficult for the detection dog to identify the precise location of the target odor source in certain species. We did not find wind speed, sand surface temperature, humidity, and the number of hours worked to have an effect on the detection dog’s performance (accuracy) for locating sea turtle eggs. Conversely, sea turtle species, clutch depth, wind direction, and ambient temperature were found to be correlated with the detection dog’s accuracy. As the clutch depth and temperature increased so did the detection dog’s location error (increased distance to clutch). This performance variation with clutch depth was expected because the target odor is unlikely to dissipate precisely straight up through the sand. We hypothesize that this effect is pronounced with higher air temperatures, which might cause the volatile scent to disperse, whereas lower temperatures keep the scent near the ground [8, 30]. As such, both increasing clutch depth and higher temperatures might make the task of locating the target odor source more challenging [8, 30]. However, the correlation presented with accuracy and ambient temperature was a weak correlation (r = 0.159). This weak correlation could be a result of the detection dog learning over time and gaining more experience.

Wind direction was also an important predictor of the detection dog’s accuracy. Other studies have noted that wind played an important role in detection dogs’ ability to locate the target odor source [24, 31–33]. Wind direction is an important factor because it will affect how easily the detection dog will pick up the target odor given the other environmental conditions (wind speed and ambient temperature) [34]. Although we did use wind direction to dictate where to begin the detection dog’s search (i.e., downwind), other factors might have affected her ability to detect the target odor source more readily, including wind speed and ambient temperature.

Although we found sea turtle species, clutch depth, wind direction, and ambient temperature to be correlated with the detection dog’s accuracy, we only found sea turtle species and wind direction to be significant predictors of the dog’s accuracy through the results of the multiple regression. Given that sea turtle species was a predictor of the detection dog’s accuracy, nesting site area (variable among species) may have contributed to a species effect. We did not measure nest area as a covariate, and we recommend that future studies of detection dog accuracy in egg detection consider this variable.

Not all studies using detection dogs have found environmental factors affecting a detection dog’s accuracy [7, 11]; therefore, these factors could be dependent on the specific detection dog or the specific type of detection work being conducted. There could also be variation in detection ability with different dog breeds and/or temperaments. More research is needed to understand how these factors might affect the accuracy of a detection dog for assisting in locating sea turtle eggs.

### Physical welfare measures

An important consideration while working with detection dogs on sea turtle nesting beaches are the extreme temperatures that often occur in these environments. According to the United States Department of Agriculture, dogs should not be exposed to temperatures above 29.4°C for more than four consecutive hours [35]. These regulations, however, are standards for housing and transportation of dogs and might not take into consideration the standards of working dogs. For example, one study examined the heat tolerance of military dogs, which revealed that they were able to withstand temperatures of 35°C up to 463 minutes (nearly 8 hours) depending on body condition [36]. Dogs are also able to adapt to certain environmental conditions, which allows them to build up a heat tolerance for specific environments needed for working standards [37, 38] such as beach surveys. Certain dog breeds are also better equipped to handle specific environments and have greater heat tolerance than other dog breeds [37, 39], which might make them better suited for detection work on a beach. These data presented in this study are the first in measuring behavior and physical environmental responses of a working dog during detection work in a beach environment. These data might provide a starting point for standards needed for detection dogs working in these conditions, similar to the data provided for detection dogs working in the desert environment [7].

## Conclusion

Detection dogs have the potential to outperform human surveyors in measures of success and reduced effort for locating sea turtle eggs. Although this study focused on one dog and handler, it provides preliminary performance results on the use of detection dogs for assisting in locating sea turtle eggs. Organizations interested in using a detection dog for sea turtle nest monitoring should first consider all the costs associated with a detection dog program and the most effective approach for using the detection dog. Additional research is needed to understand the variation in sea turtle species, environmental factors, and dog breeds. Results might also vary between dogs and handlers so further research is warranted for the use of detection dogs for sea turtle nest monitoring.

## Supporting information

S1 Appendix Training methods. Detailed training methodology for the detection dog used in this study.
(DOCX)

S2 Appendix Supporting research data. Requested data collected during this study and used for analysis.
(XLSX)
