# Supplementary material for: Use of a scent-detection dog for sea turtle nest monitoring of three sea turtle species in Florida
Source: PLoS One. 2023 Sep 13;18(9):e0290740. doi: 10.1371/journal.pone.0290740 (PMC10499246; doi:10.1371/journal.pone.0290740)
Supplement: S1 Appendix — Detailed training methodology for the detection dog used in this study. (DOCX) [file pone.0290740.s001.docx]

SUPPLEMENTAL MATERIALS

*S1. Training the Detection Dog*

Scent-detection training follows some fundamental stages, and the training methods used for Dory’s detection work provides a specific example that can be modified (e.g., different target stimulus (odor), final response behavior, search pattern, etc.) and then applied to other field-based conservation work. We recommend consulting with a professional training organization prior to acquiring a detection dog to completely understand training options specific to certain types of fieldwork. Each of the individual training phases described occurred over a two-week period, consisting of an average of 3-4 sessions a day lasting 15-20 minutes each. Success was determined when the detection dog accurately located the target stimulus at least 98% of the time with a false indication rate of less than 4%. For any of the training methods described below, the dog had the option to opt out of training and choose to participate or not participate. If the dog chose to leave the session, the dog did not get reinforced during that time and the session ended.

*S1.1 Imprinting. —*The first step for training the detection dog was imprinting her on the chosen target stimulus. The chosen target stimulus was sea turtle cloacal mucus, fluid produced by nesting sea turtles that coats the eggs during oviposition. We selected cloacal mucus because its scent is associated with freshly laid sea turtle nests, and because this method did not require collecting or sacrificing eggs; an important consideration when studying threatened and endangered species. We collected cloacal mucus from nesting loggerhead (*Caretta caretta)*and leatherback (*Dermochelys coriacea)*sea turtles using sterile swabs, which were then frozen in a 1.5mL microcentrifuge tube at -80°C for 3-6 months to preserve the scent until they were used for training purposes. Imprinting involved introducing the detection dog to the target stimulus (cloacal mucus) and associating the target stimulus with positive reinforcement (e.g., treat, toy, etc.). We used the dog’s daily diet of Purina Pro Plan dry dog food as the main reinforcer. A total of 3 cups of Purina Pro Plan was added to a training pouch at the start of the detection dog’s day to use for reinforcement. At the end of the day, any unused food was then fed to the detection dog as part of her daily diet.

Pepedogs^™^’s trainer(s) developed specific imprinting methodology that focused on using the natural instincts of the dog by allowing the dog ample time to self-discover the desired task (locate the target stimulus) and eventually achieve the final response. In other words, the dog was set up in an environment where it used its natural hunting and curiosity to encounter/discover the target stimulus on its own. Any interaction with the target stimulus was than reinforced. Imprinting occurred in several (on average, 3-4) short sessions (10-15 min) a day where the detection dog was introduced to the stimulus several times (using the previously collected mucus swabs), followed by reinforcement. This phase occurred over a two-week period. The dog was considered to have successfully imprinted on the target stimulus when the dog was consistently finding the stimulus 100% of the time during each session.

*S1.2 Simulation. —*The next step for training purposes included simulating field conditions the detection dog would experience during deployment. This step will vary depending on the type of work being performed. For this study program, Pepedogs™ created an artificial beach that included sand and other important beach characteristics such as sargassum and seashells. During the simulation phase, the lead trainer taught a search cue to indicate to the detection dog when to start searching a particular area. The search cue can be modified depending on the preference of the program. For this study program, the search cue used was “find it” or “go find it,” which indicated to the detection dog to start searching. The lead trainer also taught the detection dog to present a final response behavior, the occurrence of which indicates the location of the target stimulus to the dog handler. Similar to the search cue, the alert behavior can vary depending on the needs of the program. We selected a distinct sit behavior as the final response, which involved the detection dog pointing to the stimulus source with her nose, followed by a sit behavior and direct eye contact with the dog handler, which indicated the presence of the target stimulus. The dog handler determined the precise final response location from the initial nose point that preceded the final response (sit) behavior. Success was determined when the detection dog accurately located the target stimulus at least 98% of the time with a false indication rate of less than 4%.

*S1.3 Controlled Hides. —*After the dog/handler team had been established following simulations, controlled hide training took place. Controlled hides were designed and implemented specifically based on the target stimulus chosen. Therefore, there will be variation in how controlled hides are employed based on the specific program. For this project, controlled hides involved hiding target stimulus in diverse, controlled locations in the artificial beach environment and running the detection dog through multiple trials each day. Controlled hides provide a way for the dog handler to learn the dog’s behavior and to recognize the final response (e.g., sit) before deployment into the field. Additionally, controlled hides allowed the dog handler to perfect the timing needed for the bridge and reinforcement following a correct final response from the detection dog. After the detection dog achieved a correct final response, the handler immediately bridged the dog with “good girl” followed by a food reinforcer. The timing of the bridge and reinforcement followed closely after the correct final response (sit), so the dog associated the final response with positive reinforcement. With supervision from the lead trainer, controlled hides allowed the dog handler to establish a working relationship with the detection dog prior to performing runs without the lead trainer. Success was determined when the detection dog accurately located the target stimulus at least 98% of the time with a false indication rate of less than 4%.

During this same period, a reinforcement history was built between the detection dog and the dog handler. The reinforcement history phase included a period where the dog handler and detection dog spent most of their time together for the first two months of training, controlled hides, blind hides, and deployment. The purpose of building reinforcement history was to emphasize the role of the handler to the detection dog, to eliminate other human distractors, and to underscore the handler as the main source of receiving reinforcement for the detection dog.

*S1.4 Blind/Double-blind Hides. —*Blind hides occurred when the dog handler was aware of where the target stimulus was hidden, and therefore could properly respond to any training needed in shaping the detection dog’s final response (e.g., not reward an incorrect final response while the dog/handler relationship was being built). Double-blind hides occurred when the dog handler was not aware of where the target stimulus was hidden, and therefore reacted purely (i.e., immediate reinforcement) to the detection dog’s final response during the trial runs with no outward influence. These trials started three to four weeks prior to sea turtle nesting season, occurred in an artificial beach environment, and used the previously collected sea turtle cloacal mucus as the target stimulus.

*S1.5 Implementation/Deployment. —*Implementation involved two weeks of immersive detection work at the start of sea turtle nesting season each year in May 2017 and 2018. Each year, the lead trainer accompanied the dog/handler team for two to three days at the beginning of the season and evaluated the dog to ensure she was still able to successfully complete the task and locate the target stimulus while ignoring typical distractions. We knew the behaviors was maintained by a consistent accuracy rate of 98% and testing both dog and handler using blind hides. These two to three-day periods were considered maintenance training (detailed in section 5) for the detection dog, ensuring trained behaviors had been maintained outside of sea turtle nesting season. After the initial accuracy evaluation, the dog/handler team engaged in two weeks of immersive detection work, visiting every sea turtle nest available. During implementation, accuracy data (distance of final response to sea turtle clutch) were collected solely to measure the detection dog’s readiness for the season. The detection dog was considered ready for the season if her accuracy rate was no less than 98% presenting the final response at sea turtle nests with a false indication rate of less than 4%. After two-weeks of implementation, we deployed the detection dog into the field and started collecting data on accuracy and speed for locating the sea turtle clutch.

*S2 Dog Handler Training*

Dog handler training consisted of one week of immersive training with the lead dog trainer and the scent-detection dog. During this time, the dog handler learned about specific behavioral characteristics, behavioral needs, and care of the detection dog (e.g., high energy, enrichment needs while not working, etc.). The dog handler also learned the behavioral criteria for the trained final response (alert) behavior of the detection dog and the various behaviors associated with the dog while she was working (e.g., scenting behavior) as compared to while she was not working. The dog handler’s role included interpreting the detection dog’s behaviors to determine the target stimulus location represented by the final response, as well as other nuances of the detection dog’s behaviors. These behaviors included distraction, lack of focus, or other behaviors that could influence the accuracy of the detection dog’s initial final response location.

During controlled hides, blind hides, and double-blind hides, the lead trainer taught the dog handler how to interpret the detection dog’s behaviors and how to respond to any changes in the dog’s behaviors, reinforce positive behaviors, maintain trained behaviors, and eliminate unwanted behaviors (e.g., distracted behaviors). Unwanted behaviors were eliminated by not reinforcing them and ignoring the behaviors, giving the detection dog another option for completing the task accurately. Because dogs cue off subtle reactions from their handlers (e.g., changes in body language) that can unintentionally alter the final response, it was important that the dog handler learned to show no hesitation on reinforcing the response of the detection dog’s final response (or lack of final response), and the accuracy of the dog could be determined afterwards by digging to locate the sea turtle eggs after reinforcing the dog’s final response. The dog handler needed to reinforce all final responses given by the detection dog and control for biases that could influence outcomes. In other words, the handler avoided unintentionally reacting (e.g., changes in body language) in any way when the detection dog was near the site the handler believed the sea turtle eggs were located.

After one week of immersive training with the lead trainer, the dog handler spent one month of maintenance training with the detection dog performing a variety of controlled hides, blind hides, and double-blind hides. This training reinforced both dog handler and detection dog trained behaviors prior to deployment of the detection dog in the field. The timeframe of the dog handler training can vary depending on the complexity of the program and will be dependent on the comfort level of the dog handler prior to deployment.

*S3 Dog/Handler Relationship*

A significant factor in program success was the dog/handler relationship. As with many scent-detection programs, the number of dog handlers assigned to each detection dog was usually limited in order to create clear and consistent communication and build reinforcement history between the dog and handler.

For this program, to promote consistent communication between the dog and handler and build reinforcement history, the dog and handler remained in each other’s presence as frequently as possible throughout the first six months of the program. To increase the dog/handler relationship, the dog was kept near the dog handler during the day, walked by the dog handler, and primarily cared for by the dog handler during the bonding period. During scent-detection work, this practice ensured the dog knew primarily where all reinforcement was coming from, making distractions less likely for the detection dog. The dog/handler relationship was and should be maintained (as described above) throughout the duration of any program or the working life of the scent-detection dog.

*S4 Cross-training*

The focus of this paper was to outline the training methods for a scent-detection program using sea turtle egg location as an example, but it is important to note the specifics and possibilities of cross-training. Although cross-training has variable definitions, here it refers to teaching the detection dog how to search and final response on multiple stimuli [1].

While our detection dog was trained to locate two distinct stimuli before deployment was initiated, she did not have to discriminate between the two stimuli in the field as there was no temporal or spatial overlap. In other words, the detection dog was trained to find live gopher tortoises during one part of the year (September-April) and sea turtle eggs during another part of the year (May-August) and each occurred in different environments/habitats. Therefore, the detection dog was trained to exhibit a stimulus-independent response, meaning she presented a single final response behavior regardless of which target stimulus was identified [1]. When switching between distinct field seasons, the detection dog was reconditioned through maintenance training on the individual stimuli (gopher tortoise vs. sea turtle cloacal mucus) in a controlled setting.

Cross-training will be highly variable depending on the needs of the program. For example, a program may require the detection dog to distinguish two different stimuli at the same time and present a different final response dependent on each stimulus. Therefore, defining the goals for training the detection dog was crucial early in the project, including how many scents will be trained, if distinct cues will be used for each scent, and if distinct responses/alerts were trained for each scent. Generally, as the cognitive demand of the detection dog increases, performance declines [1-2].

*S5 Maintenance Training*

Maintenance training was a crucial step in the process to ensure the detection dog was prepared for the upcoming field season. However, maintenance training also occurred throughout the course of the fieldwork. As described, two weeks prior to the start of fieldwork, we started the detection dog on maintenance training to ready her for sea turtle nesting season. Since the two stimuli that the detection dog was trained on were unlikely to be located in the same working environment, we were able to transition the detection dog from one stimulus to another depending on the seasonal need. This training allowed the detection dog to understand that she was shifting from working on gopher tortoises (September-April) to sea turtle eggs (May-August) by reintroducing her to the other stimulus during maintenance training before deployment into the field. During sea turtle season, we also continued maintenance training by allowing the detection dog to rerun certain nests after the clutch was exposed to increase her accuracy on pinpointing clutch location and reinforce her final response behavior. This process helped maintain the trained alert response and reinforce her on certain nests that were more challenging (e.g., deeper or larger).

Maintenance training also increased the dog/handler relationship by continuing to build the reinforcement history between the dog and handler. This type of training helped the dog handler maintain their own training by understanding the cues of the detection dog while also sustaining the focus of the dog by continually interacting with the dog and providing positive reinforcement during training and fieldwork.

References

1. Lit L. Evaluating learning tasks commonly applied in detection dog training. In W.S. Helton (Ed.), *Canine Ergonomics: The Science of Working Dogs* (pp. 99-114). 2009; Boca Raton, FL: CRC Press.
2. Nippak P, Chan AD, Campbell Z, Muggenburg B, Head E, Ikeda-Douglas, CJ, Milgram NW. Response latency in *Canis familiaris*: mental ability or mental strategy? Behavioral Neuroscience. 2003; 117(5):1066–1075.
